# Factors associated with chronic kidney disease in patients with diabetes in French Guiana

**DOI:** 10.3389/fcdhc.2023.1167852

**Published:** 2023-10-25

**Authors:** Christopher Sacareau, Mathieu Nacher, Kinan Drak Alsibai, Andre Ntoutoum, Antoine Adenis, Marianne Hounnou, Marion Liebart, Clara Salasar Cardoso, Jean-Markens Aurelus, Magalie Demar, Olivier Casse, Samia Amokrane, Jean-François Carod, Nezha Hafsi, Nadia Sabbah

**Affiliations:** ^1^ Department of Endocrinology and Metabolic Diseases, Cayenne Hospital Center, Cayenne, French Guiana; ^2^ Clinical Investigation Center Antilles French Guiana (CIC INSERM 1424), Cayenne Hospital Center, Cayenne, French Guiana; ^3^ Department of Pathology and Center of biological Resources (CRB Amazonie), Cayenne Hospital Center, Cayenne, French Guiana; ^4^ Laboratory of Parasitology-Mycology, Cayenne Hospital Center, Cayenne, French Guiana; ^5^ EA3593, Amazon Ecosystems and Tropical Diseases, University of Guiana, Cayenne, French Guiana; ^6^ Department of Medicine, Ouest Guyane Hospital Center, Saint-Laurent, French Guiana; ^7^ Laboratory of Biology, Ouest Guyane Hospital Center, Saint-Laurent, French Guiana

**Keywords:** kidney disease, patient cohort, diabetes mellitus, diabetes complications, French Guiana

## Abstract

**Introduction:**

With over half of the population living under the poverty threshold, the social and health context in French Guiana is more difficult than in mainland France. The prevalence of diabetes is twice as great and end-stage renal failure is 45% higher than in mainland France.

**Objective:**

Our objective was to describe the profile of diabetic patients with chronic kidney disease in French Guiana and search for possible risk factors.

**Method:**

We conducted a multicenter cross-sectional observational study based on the CODIAM cohort (Cohort of Diabetes in French Amazonia). We analyzed 1,287 patients followed up between May 2019 and June 2021 at Cayenne Hospital, Saint Laurent Hospital, and delocalized health centers.

**Results:**

In our cohort, chronic kidney disease was present after an average of 12 years of diabetes. Compared with the French population, 41% of diabetic patients had chronic kidney disease (i.e., 12% more), and had an average age of 56 years (i.e., 10 years younger). Forty-eight per cent of these patients were obese (i.e., 7% more). Seventy-four per cent of patients were precarious and 45% were foreigners but neither was associated with chronic kidney disease, contrary to countries where the health system is not universal.

**Conclusion:**

Screening of patients with chronic kidney disease among diabetics in French Guiana remains a real challenge. Patients were younger and more obese than in other French territories. In this cohort, precariousness and immigration were not associated with the presence of chronic kidney disease. However, particular attention should be paid to hypertensive patients and those over 65 years of age, which are, with diabetes itself, the two most obvious risk factors for developing chronic kidney disease among diabetic patients in our territory.

## 1 Introduction

Diabetes is a major public health problem. The prevalence of diabetes continues to increase worldwide, mainly in developing countries. The global prevalence of diabetes increased from 4.6% in 2000 to 8.8% in 2015, and the prediction for 2040 is 10.4% (1).

Kidney disease is a severe complication of diabetes. Along with hypertension, it is one of the leading causes of chronic kidney disease (CKD) worldwide (2–5). Diabetes is an independent risk factor for cardiovascular events and mortality, but the risk is even greater in diabetic patients, especially when renal failure is advanced (6, 7).

Various risk factors for the development of CKD have been identified in people with diabetes. These include hypertension, glycemic imbalance, a prolonged course of diabetes, hypertriglyceridemia, elevated uric acid levels, and chronic inflammation (8–10). Some studies also propose the analysis of plasma biomarkers, but none are used in routine practice (11). Other studies have focused on ethnicity. An increased risk of CKD has been identified in African-American and Caribbean populations, in association with higher blood pressure, highly prevalent diabetes (12–14), and the presence of genetic variants of apolipoprotein L1 (APOL1) (15). Socioeconomic factors, such as low levels of education and income and limited access to care are also associated with an excess risk of CKD, which is over-represented in the African-American population (16).

However, these data from the literature seem difficult to extrapolate to the population and context of Guiana. French Guiana is a French overseas territory, located in South America, with a population composed of more than 22 ethnicities. It is primarily made up of Amerindians—the main occupants of French Guiana—and Creoles, who are descendants of Africans. More recently, waves of migration from Haiti and neighboring countries, such as Suriname, Guiana, and Brazil, have enriched the population (17). Much of the French Guianese population lives in a very precarious situation (18). The navigability of the health system is complex for many patients of foreign origin, which is aggravated by a low medical density and a language barrier (19). Magic–religious representations of disease and the low level of education make it difficult for patients to adhere to long-term treatments (20). More specifically, among diabetics in French Guiana, the late use of monitoring tests—such as for microalbuminuria and plasma creatinine levels—delays the diagnosis of CKD until a very advanced stage (20, 21). Despite a universal French health system, health markers are lagging behind those of mainland France:

Life expectancy is lower (19); the percentage of diabetics is twice as high as in metropolitan France, at about 10% of the adult population (17); the incidence of end-stage renal failure is 50% higher (21); the incidence of cerebrovascular accidents is one of the highest in France, particularly affecting younger patients, who have more after-effects (22); and the mortality rate for myocardial infarction seems to be higher (23). Thus, in French Guiana there is a combination of factors influencing the risk of renal damage in diabetic patients: the genetic heritage of African origin, precariousness, and the universal health system. This is scarcely described in the literature. In the present study, our first aim was not to produce another score with different variables, but instead to determine the risk factors for CKD in the population of diabetic patients in French Guiana, with the aim of better identifying possible risk groups and implementing early measures to mitigate the consequences of diabetes.

## 2 Materials and methods

We used cross-sectional data from the CODIAM cohort (Diabetes Cohort in French Amazonia) (18). These data comprise the epidemiological, clinical, and biological data of diabetics in French Guiana. We carried out a descriptive cross-sectional study based on data at inclusion. Our study was multicentric at the Centre Hospitalier de Cayenne, Saint Laurent du Maroni, and delocalized health centers and was thus representative of the population of diabetic patients in French Guiana. The recruitment of patients took place between May 2019 and June 2021, during consultations, during day or weekday hospitalizations in diabetology, and during hospitalizations in other medical services.

### 2.1 Inclusion and exclusion criteria

Inclusion criteria for the CODIAM cohort included a confirmed diagnosis of diabetes, defined as two different fasting blood glucose measurements greater than or equal to 1.26 g/L (5.5 mmol/L) or a blood glucose level greater than 2 g/L (11.1 mmol/L) at any time of day. Other inclusion criteria included being aged ≥ 18 years and signed consent to participate in the study.

The criteria for non-inclusion were subjects who refused to sign the consent form, subjects under 18 years of age, patients with gestational diabetes, patients with medical follow-up outside French Guiana, and vulnerable persons (i.e., protected adults or those unable to express their consent, pregnant and nursing women, persons deprived of their liberty, persons hospitalized without consent, persons admitted to a health or social institution for purposes other than research, and inclusions in an emergency situation).

During the medical consultation at the time of inclusion, height and weight and sociodemographic and clinical data were collected. At the end of the consultation, a blood sample was taken.

### 2.2 Variables

Chronic kidney disease was defined as either the presence of microalbuminuria greater than 30 mg/g creatinine or a creatinine clearance, estimated according to the modification of diet in renal disease (MDRD) equation, of less than 60 mL/min/1.73m^2^ at inclusion. Sociodemographic characteristics, such as nationality, education, social regime, and mother tongue, were documented. The social regimes were:

- State medical aid—*Aide Médicale d’Etat* (AME)—allows foreign patients in an irregular situation to benefit from access to health care after 3 months of residence in France.
- Universal health coverage—*Couverture Maladie Universelle* (CMU)—allows a complementary reimbursement of care not reimbursed by the health insurance.
- Long-term condition–*Affection de Longue Durée* (ALD)—is a system that provides up to 100% coverage of medical expenses for serious and/or chronic diseases requiring prolonged and expensive treatment.

The criterion of precariousness was retained if the result of the Evaluation of Poverty and Health Inequalities in Health Examination Centers (EPICES) questionnaire, corresponding to a validated precariousness score, was higher than 30.17 (24).

The criteria for complications were listed as follows:

Arterial disease was defined by abnormalities on an arterial Doppler ultrasound of the lower limbs, with a systolic pressure index of less than 0.9, or clinical signs with claudication on walking or resting ischemia.

Hypertension was considered if it was found in the medical record or during the consultation if the blood pressure was higher than 140/90 mmHg at rest.

The smoking variable was defined as active smokers. Alcohol consumption was scored as positive with one or more occasional drinks.

Retinopathy was investigated by performing a fundus examination.

Neuropathy was confirmed by a podiatric examination with monofilament test abnormalities or decreased pallesthesia, an orthostatic hypotension test, gastroparesis symptoms in favor of dysautonomia, or electromyogram results in favor of the diagnosis.

Ischemic and hemorrhagic strokes were identified.

Myocardial infarction was defined as a history of acute coronary syndrome with ST+ and non-ST+ segments.

The diabetic foot was defined by the presence of an infection, ulceration, amputation of the feet, or deformity in favor of a Charcot foot.

We measured 27 biological parameters usually used in the follow-up of diabetic patients. In our study, we analyzed levels of hemoglobin, glycated hemoglobin, C-reactive protein (CRP), high-density lipoprotein (HDL) and low-density lipoprotein (LDL), triglycerides, plasma creatinine, urinary creatinine, and urinary albumin.

### 2.3 Primary endpoint

The primary endpoint was the presence of CKD in diabetic patients.

### 2.4 Secondary endpoint

The secondary endpoint was the assessment of clinical, biological, and sociodemographic characteristics of patients with CKD.

### 2.5 Statistical analysis

The statistical analysis was performed using STATA® (StataCorp LLP, College Station, TX, USA). A descriptive analysis of the population was performed according to the presence or absence of CKD. Quantitative data are expressed as means and standard deviations. Qualitative data are reported as numbers and percentages. Comparisons of quantitative variables between groups were made with parametric or non-parametric tests according to the distribution of the variables. Comparisons of qualitative variables between groups were made using the chi-squared test. The level of significance retained was 5%. The multivariate association between CKD and other diabetic complications was done by a logistic regression. Due to the large number of variables, we could not include all variables in the multivariate analysis. In addition, many data were missing at random, which significantly reduced the number of patients in the analysis. Our modeling strategy was not to obtain the most pragmatic model to make a prediction but simply to be pragmatic and include all variables with few missing values to unmask for confounding. We computed the sensitivity, specificity, and positive and negative predictive values.

### 2.6 Ethics and protection of persons

All patients included in the study were informed about the anonymous use of their data for research and signed a consent form.

Data analysis was subject to analysis protection, registration at the hospital analysis center, and a declaration of compliance with MR003. The protocol was approved by the South-East Personal Protection Committee in Clermont-Ferrand (No 2020/CE 04). Our study was in accordance with the General Data Protection Regulation and the French Data Protection Act.

## 3 Results

**Figure 1:**
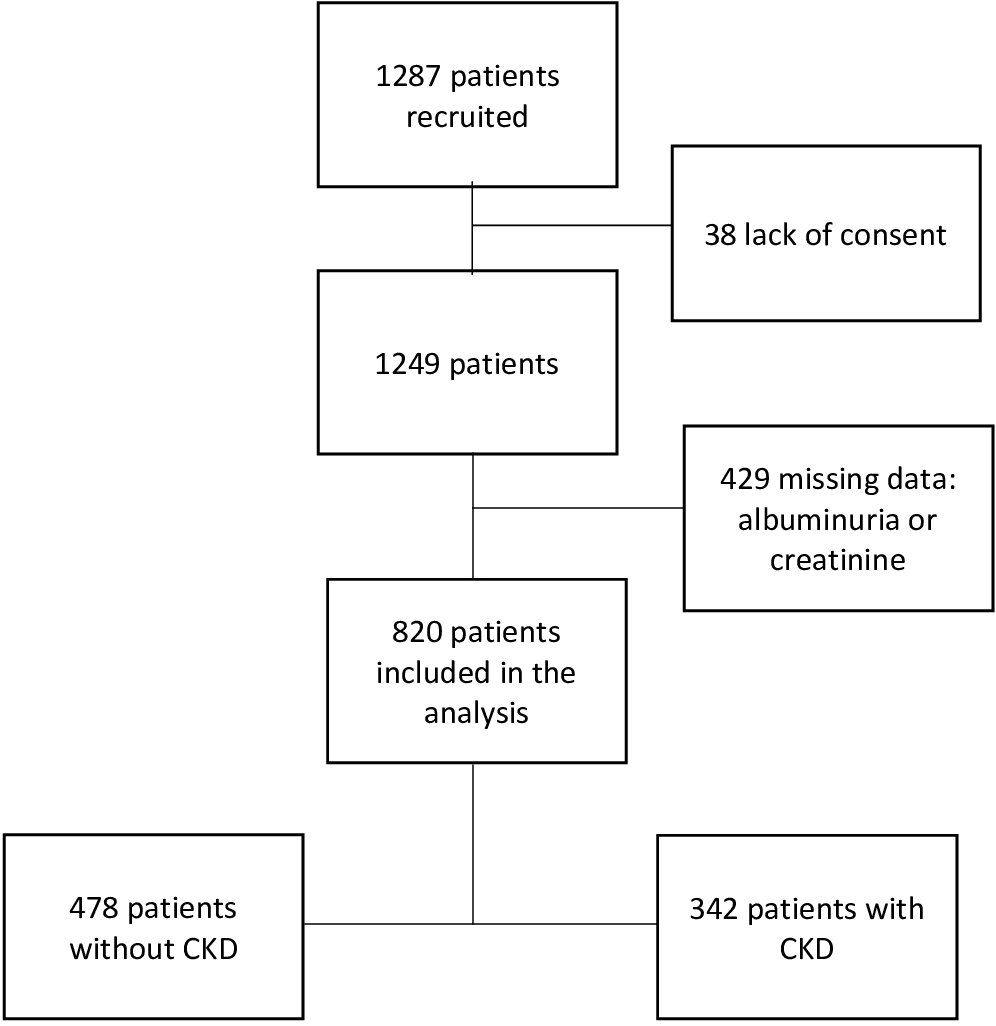
Flow chart.

The study involved 1,287 patients. A total of 1,209 patients were from the Cayenne Hospital, 73 from the West Guiana Hospital, four from Grand-Santi, and one from Maripasoula (delocalized health centers). Thirty-eight patients did not sign a consent form and were excluded from the analysis. The MRC variable of interest was available for 820 of the patients, which represented 429 missing data (493 missing data for albuminuria and 96 missing data for renal function). We made two groups: presence of CKD in 342 patients (42%) and absence of CKD in 478 patients (58%) (**Figure 1**).

The CKD variable has been summarized in **Table 1** below.

**Table 1:** Description of CKD.

Estimated renal function (*N* = 805), *n* (%)	
Stage 1 or 2	659 (82)
Stage 3	98 (12)
Stage 4	29 (4)
Stage 5	14 (2)
Albuminuria (*N* = 756), in mg/g creatinuria, *n* (%)	
No	510 (68)
Microalbuminuria	189 (25)
Macroalbuminuria	57 (7)

Chronic kidney disease was primarily represented by patients with micro or macro albuminuria (32%). Renal failure from stage 3 onwards occurred in 18% of the patients. Severe and end-stage renal disease was even less frequent, occurring in only 4% of the sample. In the study population, 108 patients (13%) had hyperfiltration, i.e., an estimated glomerular filtration rate (eGFR) according to the MDRD equation of > 130 mL/min/1.73m^2^.

The distribution of clinical, sociodemographic, and biological data of the main data is described in **Table 2**.

**Table 2:** Description of clinical, sociodemographic, and biological data according to CKD status.

	CKD		
	Absence	Presence	
	(*N* = 478; 58%)	(*N* = 342; 42%)	
Age (years)	53.5 (13.3)	60.0 (13.5)	***p* < 0.0001**
Sex			*p* = 0.15
Female	263 (61%)	171 (39%)	
Male	215 (56%)	171 (44%)	
Body mass index (kg/m^2^)	30.4 (6.6)	30.8 (6.5)	*p* = 0.36
Obesity	225 (48%)	160 (48%)	*p* = 0.94
Tobacco use	52 (12%)	32 (11%)	*p* = 0.60
Alcohol use	222 (48%)	126 (39%)	***p* = 0.01**
Type 2 diabetes	420 (95%)	314 (95%)	*p* = 0.82
Hypertension	301 (64%)	290 (86%)	***p* < 0.0001**
Hemoglobin	12.9 (1.4)	12.1 (1.9)	***p* < 0.0001**
Anemia	152 (34%)	182 (56%)	***p* < 0.0001**
HbA_1c_	8.7 (2.3)	8.7 (2.1)	*p* = 0.63
Insulin treatment	122 (30%)	90 (28%)	*p* = 0.57
Treatment by: Angiotensin-converting enzyme inhibitor Angiotensin II receptor antagonist	185 (39%)	208 (60%)	***p* < 0.0001**
Duration of diabetes	7.5 (8.3)	12.6 (10.9)	***p* < 0.0001**
Lipids			
LDL-cholesterol	2.62 (0.89)	2.47 (1.07)	***p* = 0.04**
HDL-cholesterol	1.20 (0.38)	1.18 (0.47)	*p* = 0.64
Triglycerides	1.45 (0.95)	1.67 (1.44)	***p* = 0.001**
Complications:			
Retinopathy	48 (12%)	60 (21%)	***p* = 0.0005**
Arteriopathy	18 (3.8%)	41 (12%)	***p* = 0.0005**
Neuropathy	53 (11%)	54 (17%)	***p* = 0.03**
Diabetic foot	11 (2%)	26 (8%)	***p* = 0.0039**
Myocardial infarction	46 (12%)	34 (12%)	*p* = 0.90
Stroke	87 (22%)	58 (20%)	*p* = 0.50
Therapeutic education	341 (71%)	233 (68%)	*p* = 0.88
Nursing assistance	158 (33%)	148 (43%)	***p* = 0.042**
Social security			
No	22 (5%)	9 (3%)	*p* = 0.14
State medical aid	34 (7%)	14 (4%)	*p* = 0.06
Universal medical coverage	206 (43%)	163 (48%)	*p* = 0.19
Long-term condition	347 (73%)	263 (77%)	*p* = 0.16
Precariousness	298 (73%)	222 (77%)	*p* = 0.30
Foreign nationality	217 (45%)	141 (44%)	*p* = 0.72
Post-high school education	89 (20%)	48 (15%)	*p* = 0.10
Spoken French			*p* = 0.22
Does not speak or speaks poorly	25 (5%)	24 (7%)	
Quite good or fluent	444 (95%)	299 (93%)	
Written French			*p* = 0.41
Good or a little	348 (75%)	231 (72%)	
Poorly or not at all	116 (25%)	88 (28%)	

For quantitative variables, the data represent the medians and standard deviations.

For categorical variables, the data represent the numbers and percentages.

Bold values highlight statistical significance.

Ninety-five per cent of the patients in the study had type 2 diabetes, with no difference in distribution between the two groups. The anthropomorphic characteristics—body mass index (BMI) and obesity—were not different either. The rate of smoking was low (12% of the population); alcohol consumption was higher (44% of the population). Patients with CKD were more hypertensive. Hypertension was found in 72% of patients.

The patients in the CKD group were significantly older, with longer term and more complicated diabetes. Microangiopathy complications—retinopathy and neuropathy—were more common when CKD was present.

Macroangiopathy complications had a variable distribution between groups: arteriopathy was more prominent in patients with CKD, in contrast to stroke and myocardial infarction (MI), for which no difference was observed between the groups. The sociodemographic data of the sample showed a large proportion of patients of foreign origin (45%), comprising 24% Haitian patients. The other, less represented nationalities were Brazilians, Surinamese, and Guyanese. No association was found between nationality and the presence of CKD. Precariousness was very important in the study (74% of patients) and was not associated with CKD. We did not find any difference in the level of schooling or in the oral or written mastery of the French language. Therapeutic education was not different between the two groups, whereas home nursing assistance was more prominent in patients with CKD. Long-term conditions were found in 74% of patients.

The patients with CKD benefited more from nephroprotective angiotensin-converting enzyme inhibitor (ACE I)/angiotensin II receptor antagonist (ARA2) therapy. However, 33% of patients with hypertension and microalbuminuria did not receive treatment despite an indication (**Table 3**).

**Table 3:** ACE I/ARB2 intake by indication.

	Treatment with nephroprotective ACE Is/ARA2, *n* (%)	
	Yes	No
Hypertension	368 (63)	223 (37)
Hypertension + albuminuria	134 (66)	67 (33)

Data from the multivariate analysis between disease and risk factors for chronic kidney disease our population are listed in **Table 4**.

**Table 4:** Logistic regression analyses.

Chronic kidney disease (*N* = 670)	Odds ratio	95% CI	*p*-value
Age over 65 years	**2**	1.4–2.9	**< 0.00**
Male gender	1.29	0.9–1.8	0.12
High blood pressure	**2.45**	1.6–3.6	**< 0.00**
Obesity	1	0.7–1.4	0.98
Precariousness	1.32	0.9–2.0	0.17
Foreign nationality	0.80	0.6–1.2	0.25
Does not speak French	1.76	0.8–3.8	0.14
AME	0.55	0.2–1.3	0.20
CMU	1.06	0.7–1.5	0.74
ALD	0.86	0.6–1.3	0.51

The association between CKD and major risk factors in the multivariate analysis.

Bold values highlight statistical significance.

**Figure 2:**
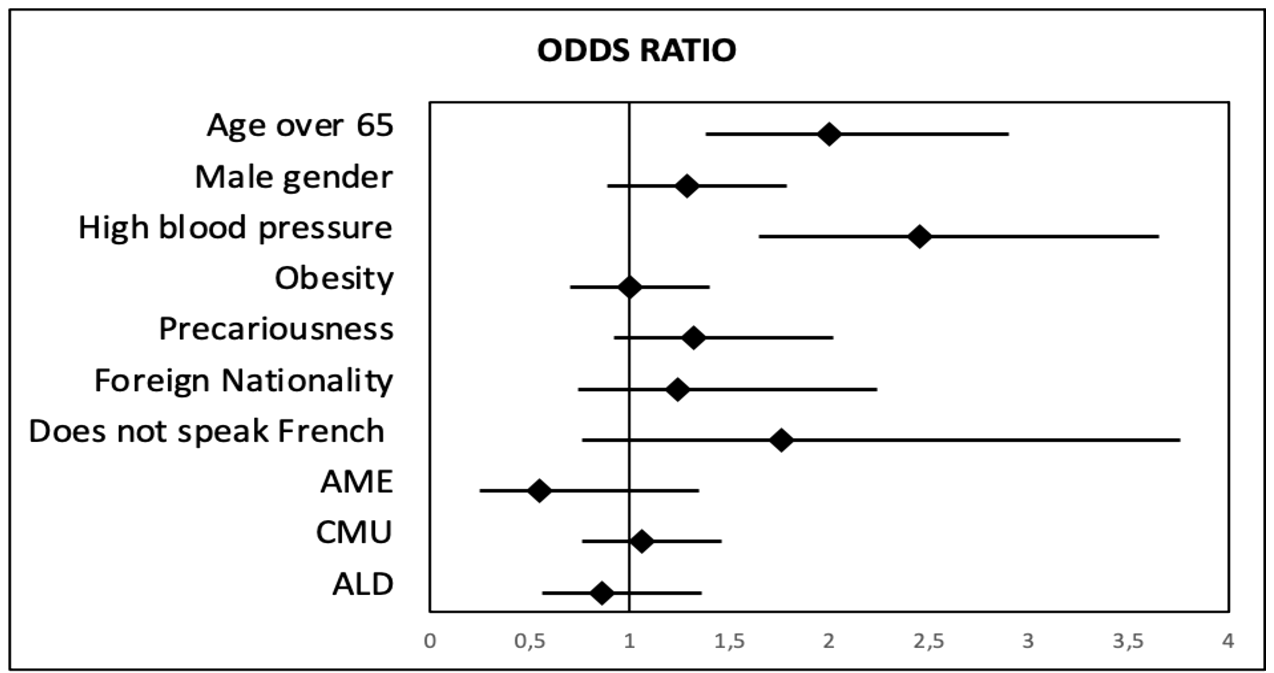
Odds ratio between chronic kidney disease and risk factors in multivariate analysis.

After the multivariate analysis, we found an association with hypertension and being over 65 years of age. No sociodemographic data were significant in the multivariate analysis (**Figure 2**).

In terms of prediction, the multivariate analysis yielded the following measures: sensitivity 37%, specificity 81%, positive predictive value 59%, and negative predictive value 65%.

## 4 Discussion

The proportion of patients with CKD in our study was 42%, which is higher than the national average of 29% but comparable to the world average of 40% (with large variations according to state) (25–27). This rate was probably underestimated due to the absence of CKD data in 34% of the patients in the sample, mainly due to the absence of urine sampling, which requires the need to urinate when going to the laboratory. In addition, 33% of the non-proteinuric patients in our population were taking an ACE I or ARB2 that could mask proteinuria and erroneously classify them as CKD free (28). The sociodemographic data from Guiana do not explain the percentage of CKD. The precariousness in our study was important—relevant for 74% of patients—and was not associated with CKD. Other studies in French Guiana (18), and in mainland France (29), did not find any association either, contrary to the USA where poor diabetic patients present more CKD (30). Precariousness in French Guiana is therefore less of an obstacle to care and monitoring of chronic pathologies. In fact, 74% of the patients in our study were on ALD, allowing them to receive 100% reimbursement of care. Better still, patients with CKD were more likely to be monitored by a home care nurse (without any link to insulin use). Our data are supported by a study in French Guiana in which poor patients had better home nursing follow-up, more therapeutic education, and more frequent registration in ALD for diabetic patients (18). Nationality was not associated with CKD in our study. In our population, 45% of the patients were of immigrant origin, close to the 40.5% observed in the Cayenne population, which constituted the majority of recruitments (19). In our population, contrary to our initial assumptions, the level of education or poor French-language skills were not associated with the presence of CKD. This is contrary to the literature, which shows that the most educated patients are the most apt to evolve in the care pathway and adhere to care (31). Moreover, we did not demonstrate the role of therapeutic education in the prevention of CKD.

Despite this, the situation in French Guiana is complex, as 30.9% of patients have refused care for financial reasons, with the majority of patients having an immigrant background (32). However, the main reason for not seeking care is the long waiting times for consultation due to the low medical density. Social factors did not explain the higher percentage of CKD in our population. Diabetic patients were 10 years younger, with an average age of 56 years compared with 66 years in France (25). A study in the USA compared the average ages of African-American diabetic patients—younger than white patients, but with an average age of 65 years. This age difference was not related to the prevalence of type 1 diabetes—occurring at a young age—which is similar to that in France (33). We did not find any obvious factor explaining this age difference. However, patients with CKD were older with a longer duration of diabetes, which was expected (34).

The prevalence of obesity was higher in our population: 48% of patients compared to 41% in mainland France. However, obesity—recognized as a cause of microalbuminuria (35)—was not associated with CKD, especially in the multivariate analysis.

Hypertension was found in 72% of the study population. We did not find any prevalence of hypertension in diabetics in French Guiana in the literature, but these data are comparable with those found in France, where there is a nearly identical prevalence of around 70% (36). On the other hand, hypertension was more frequent in the CKD group, as in other studies (37). This association remained in the multivariate analysis. Although the multivariate model’s sensitivity to predict renal failure was low (37%), its positive predictive value was 59% and its specificity was 81%. The use of ACE Is or ARB 2 was also more frequent in the CKD group, in favor of nephroprotection. However, improvements are needed because 33% of hypertensive patients with microalbuminuria did not benefit from nephroprotective treatments despite level 1 recommendations.

The sex difference was not associated with an increased risk of CKD. Many studies have been done, but the trend, especially in meta-analysis, is toward an increased risk of CKD and end-stage renal disease in women, with a smaller sex difference in the African-American population (38, 39).

The literature shows a lower level of glycated hemoglobin in patients with chronic renal failure, related to anemia (40). However, the levels of glycated hemoglobin were not different between our groups despite a more severe anemia in chronic renal disease. This result could be due to poorer diabetes control in the CKD group. Regarding complications associated with diabetes, not surprisingly, diabetic retinopathy, diabetic neuropathy, and arteriopathy are associated with CKD in our study as in the literature (41–43). However, stroke and MI were not associated with CKD in our population, contrary to what might have been expected (44, 45), with no obvious explanation.

Our study has limitations. Chronic kidney disease was defined by structural or functional renal abnormalities lasting more than 3 months. We had only one measurement of microalbuminuria or creatinine. Nevertheless, recruitment took place under stable conditions, mainly in consultations and the day hospital. Even in weekday hospitalization, patients were hospitalized mainly for glycemic imbalance or complications, and had stable kidney function. Patients admitted to the medical ward were not admitted for acute renal failure or urinary tract infection. We therefore believe that these results can be extrapolated to CKD.

The multicenter recruitment of 1,287 patients allowed us to extrapolate our results to the Guyanese population, even if the majority of the recruitments took place in Cayenne.

Our study was original, as few studies focus on CKD in the West Indies and French Guiana region, unlike end-stage renal disease, which is well studied in the Renal Epidemiology and Information Network (REIN) registry (21). In 2014, only one study evaluated screening for proteinuria by general practitioners of type 2 diabetics in the overseas departments. This study found similar data, with proteinuria in 45% of patients, especially younger ones (46).

Despite a higher rate of chronic renal disease among diabetics, patients do not progress more toward end-stage renal failure in French Guiana. As in mainland France, the percentage of end-stage renal failure of diabetic origin is between 20% and 25% (21).

## 5 Conclusion

The screening of patients with CKD among diabetics in Guiana remains a real challenge. In 2019, only 42.9% of diabetics had annual creatinine clearance and microalbuminuria determinations. In our cohort, CKD among diabetics was more frequent than in mainland France. Precariousness and immigration were not associated with the presence of CKD, with a compensatory role of the health system, unlike in the USA. No other obvious factor was found to explain this difference. However, particular attention should be paid to hypertensive patients and those over 65 years of age, which are the two most obvious risk factors for developing CKD in our territory.

## Data availability statement

The original contributions presented in the study are included in the article/supplementary material. Further inquiries can be directed to the corresponding author.

## Ethics statement

The study protocol was approved by the French regulatory authority, the Commission Nationale de l’Informatique et des Libertés (CNIL). The protocol was approved by the Comité de Protection des Personnes (Committee for the Protection of Persons) Sud-Est de Clermont-Ferrand (Nos ref: 2020 /CE 05). All patients provided written informed consent.

## Author contributions

Conceptualization: CS, MN, AA, and NS. Data curation: CS, MN, KD, AN, AA, MH, ML, CSC, J-MA, OC, SA, NH, and NS. Formal analysis: SC, NM, and NS. Investigation: SC, MN, KD, AN, AA, MH, ML, J-MA, OC, SA, NH, and NS. Methodology: CS, MN, AA, OC, and NS. Project administration: CS, MN, AA, CSC, J-MA, NH, and NS. Resources: MN, DM, and NS. Software: CS, MN, AA, and NS. Supervision: MN, and NS. Validation: CS, MN, KD, AN, AA, MH, ML, CSC, J-MA, MD, OC, SA, J-FC, NH, and NS. Writing: CS, MN, and NS. All authors contributed to the article and approved the submitted version.
